# Nephroprotective Potential of Lyophilized *Grewia asiatica* Powder Against Renal Biomarkers and Inflammation In Vivo

**DOI:** 10.1155/jnme/3726752

**Published:** 2025-04-19

**Authors:** Saima Latif, Muhammad Sohaib, Sanaullah Iqbal, Muhammad Hassan Mushtaq, Muhammad Tauseef Sultan

**Affiliations:** ^1^Department of Food Science & Human Nutrition, University of Veterinary & Animal Sciences, Lahore 54000, Punjab, Pakistan; ^2^Department of Epidemiology & Public Health, University of Veterinary and Animal Sciences, Lahore 54000, Punjab, Pakistan; ^3^Department of Human Nutrition, Bahauddin Zakariya University, Multan, Punjab, Pakistan

**Keywords:** cisplatin, *Grewia asiatica*, health benefits, neoplasm, nephroprotective, serum creatinine

## Abstract

**Introduction:** Phalsa (*Grewia asiatica*) fruit is known for its rich nutritional profile and diverse pharmacological properties such as antioxidants, anti-inflammatory, and anti-cancer, making it a promising contender for preventive measures against cisplatin-induced acute kidney injury (AKI) in living organisms.

**Material and Methods:** In the present study, rats were provided with different levels of lyophilized *Grewia asiatica, i.e.,* 200, 300, and 400 mg/kg body weight along with control, fed on the basal diet. After trial completion, blood serum samples of rats subjected to renal biomarkers, hematology, and liver function tests, interleukin-6 (IL-6), whereas enzymes (alanine aminotransferase (ALT); sodium oxide dismutase, and glutathione) for kidney tissues along with photomicrographs for kidney tissue damage were measured.

**Results:** The findings revealed that lyophilized *Grewia asiatica* provision effectively reduced renal biomarkers, blood urea nitrogen, and creatinine with AKI in the rats as well as treatments demonstrated significant improvements in antioxidant activity by reducing malonaldehyde levels and increasing the activity of glutathione, catalase, and superoxide dismutase in groups treated with dosages of 300 and 400 mg/kg powder.

**Conclusion:** *Grewia asiatica* exhibited remarkable hepatoprotective properties by decreasing ALT and displayed anti-inflammatory properties, as evidenced by a substantial decrease in interleukin-6 serum levels. The study findings also added valuable insight into the multiform nephroprotective reverberation of lyophilized phalsa powder, emphasizing its plausible protective use in reducing cisplatin-induced nephrotoxicity.

## 1. Introduction

The kidney is an essential organ responsible for eliminating metabolic waste products from blood into urine to maintain homeostasis in human body. Acute kidney injury (AKI) is critical, often life-threatening condition characterized by a sudden decline in kidney function, leading to impaired fluid and electrolyte balance along with the excretion of endogenous waste materials in humans. Additionally, drug-induced nephrotoxicity is the leading cause of AKI in hospitalized patients around the globe [1]. It is estimated that > 13 million people in the world is affected by AKI [2], whereas one-third of AKI patients need ongoing dialysis after discharge from hospital setting that can decrease the quality of life [3]. It is also established that even mild or reversible AKI can result in serious consequences like deaths in humans [4].

Cisplatin (CP) is platinum-based drug used to treat malignancies; it can induce apoptosis in renal tissues via the stimulation of reactive oxygen species (ROS) resulting in AKI [5]. Nephrotoxicity is a limiting factor in its use, resulting in a progressive decline in the patients' renal capacity of around 25%–40% that can emerge as AKI, renal tubular dysfunction, hypomagnesemia, low calcium levels, high urea levels, and chronic kidney disease [6]. The nephrotoxicity involves intricate mechanisms such as oxidative stress, inflammation, direct cellular damage within renal tubules, and DNA damage [7]. This antineoplastic drug accumulates more in renal tissues than other organs, and its concentration in proximal tubular cells is about five times of that in blood, resulting in high nephrotoxicity. The accumulation of CP in mitochondria is due to the positively charged products of CP produced, being attracted by the negatively charged mitochondria, particularly renal tubules, as they have abundant mitochondrial matrix [8]. The decrease/prevention of drug side effects is the main concern for patients subjected to treatments and require taking drugs for a long period or permanently. In this context, healthy diet containing dietary antioxidants may represent a viable strategy for preventing CP-induced nephrotoxicity [9].

*Grewia asiatica* L., commonly known as phalsa, is distributed across tropical and subtropical regions of Asia, Africa, and western Indian ocean islands. It is native to the Indian subcontinent, including Pakistan, India, Bangladesh, Nepal, and Sri Lanka, and extends into the East and West Himalayan regions. The species is also found in parts of Indo-China, such as Cambodia, Laos, Myanmar, Thailand, and Vietnam, as well as in Iran. Additionally, it has been introduced to Malesia, the Philippines, and parts of Australasia, including Queensland, Australia. Its adaptability to warm climates and semi-arid conditions supports its widespread cultivation and naturalization. The botanical authority “L.” refers to Carl Linnaeus (1707–1778), who first formally described the species [10].

*Grewia asiatica* fruit contains beneficial compounds such as phenols, anthocyanin, ascorbic acid, carotene, and vitamin A along with sodium, potassium, magnesium, and cobalt [11]. With its rich array of phytocompounds, tannins, vitamins, flavonoids, amino acids, and anthocyanins, this fruit is truly a wonder of nature [12, 13]. *Grewia asiatica* demonstrated remarkable properties owing to phenolics and antioxidants that can help to provide kidney-protective effects, which could help prevent the development of renal disorders as well as kidney stone formation [14]. *Grewia asiatica* showed a protective activity against the time-administered dose of CCL_4_ due to its potent antioxidant and immunomodulatory like anthocyanidins, β-sitosterol, stigmasterol, and naringenin [15]. The presence of biopeptides like phenolics and flavonoids particularly played these roles, which also suggested fruit utilization for designing dietary therapies to protect human health against maladies [16]. Casticin is a flavonoid derived from various plant sources, known for its potent anti-inflammatory, anti-cancer, neuroprotective properties, and antioxidant properties. A research study reported therapeutic capability of casticin against CP-induced renal damage, and results reported casticin to have a remarkable defensive role against oxidative stress and inflammation, which are principal representatives of CP-induced renal damage [17].

The lyophilization process not only bolsters stability and prolongs shelf life but also addresses potential concerns related to bioavailability. This method provides a comprehensive utilization of the fruit, enabling the synergistic effects of its diverse compounds. The research endeavors to offer valuable insights into the protective mechanisms of phalsa, against CP-induced nephrotoxicity. Lyophilization of fruits offers multiple benefits for nutritional composition as well as extension of fruits' shelf life with enhanced flavor and color attributes when compared with conventional methods. The reason is that lyophilization is done at lower temperatures, which help in preserving heat-sensitive nutrients, vitamins, and antioxidants that will be lost in conventional drying. The freeze-drying inhibits microbial growth and reduces the risk of spoilage and contamination in the fruits and their products [18]. Considering the scenario, the present investigation delves into the potential nephroprotective effects of the lyophilized *Grewia asiatica* fruit pulp powder in alleviating CP-induced nephropathy using rat animal model. By selecting the lyophilized form over extracts, the present study aimed to safeguard inherent composition and bioactive compounds present in fruit along with *Grewia asiatica*, renowned for its antioxidant and anti-inflammatory properties and is being explored as a potential safeguard against CP-induced kidney damage along with establishing a basis for subsequent investigations in formulating therapeutic strategies for chemotherapy-induced kidney injury.

## 2. Materials and Methods

The present study was conducted at the Department of Food Science & Human Nutrition, University of Veterinary and Animal Sciences Lahore aimed to determine the potential of lyophilized *Grewia asiatica* powder using rodent modeling.

### 2.1. Chemicals, Reagents, and Experimental Material Procurement

All chemicals including casticin, CP, and reagents utilized in the study possess the highest purity grade and were acquired from Sigma Aldrich (Merck, Darmstadt, Germany). The kits required for the analysis of creatinine (Enzymatic UV) Cat. No: CR8317 and serum Urea (Enzymatic Kinetic) cat UR8334, Aspartate Aminotransferase (AST) (UV) (Mod. IFCC) Cat. No: AS8306, alanine aminotransferase (ALT) (UV) (IFCC) Cat No: AL3801, Total Protein (Biuret) Cat. No: TP245, Albumin (BCG) Cat No: AB 8301, all mentioned kits were commercially available by (Randox Laboratories Ltd. Crumlin, County Antrim, BT29 4QY, United Kingdom). While interleukin-6 (IL-6) Kits were purchased from Boniva Company (Beijing, China). The feed ingredients (corn starch, corn oil, wheat bran, casein protein, cellulose, vitamins, and minerals) for the rat diet were made available from the local market.

### 2.2. Preparation of Phalsa Extract for Bioactive Compound Identification Using Gas Chromatography–Mass Spectrometry

For the determination of bioactive compounds, the phalsa fruit pulp sample was mixed in a blender followed by sieving to prepare the extract of the fruit samples. Accordingly, 250 g of sieved phalsa pulp sample together with 12 mL of 5% (w/w) of CaCl2 solution was homogenized in a blender for 2 min. Afterward, 4 g of homogenized fruit pulp sample was added with 50 mL of dichloromethane soaked in a 500-mL flask. Then, the samples were stirred at 4°C for 30 min under a nitrogen gas. Afterward, the samples were centrifuged at (9000 rpm) for 15 min and the temperature was maintained at 4°C. Afterward, the organic extract in the supernatant under a nitrogen stream was collected and the extract was stored at −20°C in a glass vial equipped with a Teflon-lined cap before the analysis. Afterward, gas chromatography–mass spectrometry (GC–MS) analysis of phalsa extract was carried out in a combined 7890A gas chromatograph system (Agilent, USA) and mass spectrophotometer 5975 C, equipped with a DB-5 MS fused silica column (5% phenyl methyl siloxane 30.0 m length and 0.25 mm, 0.25 μm film thickness) employed for GC–MS analysis coupled with 5675C following the procedure of [19]. A triple-axis detector was used for inert MSD. The helium gas was utilized as a carrier gas and modified to fit the velocity of column. A pressure: 16.2 psi; temperature at interface: 250°C; 1.8 mm; and a single injector (μL) in split mode with a 1:50 split ratio and a 300°C injection temperature. The column temperature began at 36°C for 5 min and then increased to 4°C/min and temperature was increased at a rate of 20°C per minute till 250°C was maintained for 5 min. The elution time was 35 min, and the percentage quantity of every component was determined, by measuring its average peak area in relation to the entire area.

### 2.3. Preparation of Lyophilized *Grewia asiatica* Powder

For the study, fresh *Grewia asiatica* fruit was procured from Nagana fruit farm, Multan, Pakistan. Before harvesting fresh fruits, they were carefully checked for commercial maturity, color uniformity, and physical contaminant adherence and sorted for uniformity in size. After harvesting, fruits were washed and rinsed thoroughly with tap water to remove adhered particles and impurities. Afterward, they were left in open trays to dry water droplets followed by placing at an ambient temperature of 25°C. Then, using a stainless-steel knife, the flesh was separated from seed and kept in a freezer (HDF-325INV-Haier) at −18°C until frozen. Afterward, the fruit pulp was spread uniformly in different Petri plates and placed in a freeze-dryer (Alpha 1-4LD) at a temperature of −70°C to dry the product followed by grinding to a constant powder particle size. The pulp was ground into a fine powder using a commercial electric grinder (NM-8300) followed by placing in air-tight, aluminum foil-sealed bags for further analysis and rat trial application.

### 2.4. Ethical Approval

All methods and procedures undertaken for experimental protocol in rat trial were approved by the Animal Ethics Committee, University of Veterinary and Animal Sciences, Lahore. The ethical certificate was duly issued, detailing the approved trial and analysis procedures duly issued bearing REF. No. DR/206; dated: 15 May 2023.

### 2.5. Experimental Design

For the study, (42) male Wistar rats were categorized randomly into seven equal groups, with six rats in each group. The details of the rats provided with dietary lyophilized phalsa powder are mentioned in (Table 1).

### 2.6. Rat Trial, Animal Housing, and Management

The rats were provided with standard diet and *ad libitum* access to water. Forty-two male albino Wistar rats, aged 12 weeks, weights ranging from 200 to 250 g sourced from the animal breeding facility at Bahauddin Zakariya University Multan, were used in the animal trial. The animals were accommodated in a controlled environment room with temperature at 23 ± 2°C, relative humidity of 55%, and light–dark cycle of 12 h that facilitate rats' growth.

### 2.7. Induction of Acute Renal Injury in Rats

To induce ARI, selected rats were intraperitoneally injected with 5 mg/kg CP, cis-diammine platinum II dichloride (1 mg/mL, Sigma, USA) on the 28^th^ day of the trial by following the procedure [20], whereas *G*_0_ group was fed with casticin and injected with CP as mentioned by [21]. *G*_0_ and *G*_3_ were intraperitoneally injected with normal saline only. To check the incidence of AKI, blood samples drawn from rats in control and CP control groups (72 h after CP injection) from tail tip and serum creatinine level were determined. Rodents were considered to have AKI if their creatinine level exceeded 0.8 mg/dL. At the end of treatments, on the 33^rd^ day, rats were anesthetized with ketamine (75 mg/kg) and xylazine (5 mg/kg) given in combination till the completion of trial.

### 2.8. Monitoring Parameters During Study

The rat's weight (baseline and termination) during the experimental trial was measured using an electronic balance (Model: UX 420H, Shimadzu-Japan) and recorded weights subjected to an austere process of triplicate measurements by following the protocol of [22].

### 2.9. Samples Collection

In this investigation, blood withdrawal from rats was assiduously conducted on the third day following the administration of CP to assess the potential induction of AKI. On the fifth day after induction of nephropathy, rats were ultimately sacrificed by the trained animal handler and blood was drawn from the inferior *vena cava* and collected in separate vials for blood and serum for further analysis. The collected blood samples were centrifuged at 3500 rpm for 15 min and placed at 4°C to isolate plasma followed by subsequent storage at −18°C for biochemical investigations. Moreover, the rats' kidneys were removed, cleaned in ice-cold saline solutions, and blotted dry with filter paper before being weighed. Post-extraction, a thorough cleansing of kidneys in ice-cold saline solution was performed to minimize potential artifacts. A portion of kidney tissues was meticulously preserved in 10% neutral buffered formalin solution, and this preservation allows for the microscopic examination of tissue architecture and cellular morphology. The remaining kidney tissues underwent swift dissection on an ice-cold plate, followed by rinsing with ice-cold normal saline solutions. Afterward, these tissues were promptly stored at −80°C in an ultra-low temperature freezer (MDF-U33) to ensure the preservation of cellular structures for the analyses [23].

### 2.10. Serum Analysis

After rats' venipuncture, blood samples were collected and placed in ethylenediaminetetraacetic acid (EDTA) tubes and then transported to the laboratory for complete blood count (CBC) analysis within 24 h. For CBC analysis, 15 μL of blood was loaded in the whole blood mode in a Mindray automated vet blood analyzer following the protocol by [24] with temperatures set at 10°C∼30°C, humidity 20%∼85%, and air pressure about 70–106 kPa, and the analysis was initiated for parameters including red blood cells (RBCs) (10^6^/L), Hb(g/L), hematocrit (HCT) (%), and white blood cells (WBCs).

The elecsys IL-6 assay was executed on cobas e411 analyzers (Roche Diagnostics International AG Rotkreuz, Switzerland) to analyze the IL-6 level by following the procedure of [25]. Accordingly, 30 μL of blood samples treated with biotinylated monoclonal IL6-specific antibody in the first stage. Afterward, a sandwich complex was formed with a sample antigen by monoclonal IL6-specific antibody labeled with a ruthenium complex and streptavidin-coated microparticles during the second incubation. Then, the reaction mixture was aspirated into a measurement cell, where microparticles were magnetically trapped on the electrode's surface. Unbound chemicals were removed, and a photomultiplier was used to measure chemiluminescent emission caused by applying a voltage to the electrode. The elecsys IL-6 assay demonstrated a range of 1.5–5000 pg./mL, and the analyzer automatically estimates the analyte concentration in pg./mL for each analyzed sample.

The biochemical analyses were conducted using an RX Daytona Plus analyzer to assess total protein, albumin, alkaline transferase (ALT), creatinine, and urea by following the procedure of [26]. For the analysis, loading a carousel with barcoded reagents ranging from 20 to 70 mL allowed for flexible assay configurations. Sample positions and dedicated control positions on the analyzer are barcoded for meticulous sample tracking. The programmable chemistry parameters were set for each specific assay to ensure accuracy. The dual five-speed stirrer, preset based on assay parameters, facilitated optimal mixing. The instrument utilized dedicated micropipettes with liquid sensors and crash detection for precise sample handling and minimized carryover, whereas the RX Daytona Plus features were determined using a 12-stage washing process to maintain instrument cleanliness. Afterward, 72 semi-permanent cuvettes, each with an onboard cuvette checking function, ensured accuracy throughout the analytical process.

### 2.11. Antioxidant Enzyme Assays for Kidney

The glutathione (GSH) content of kidney tissue samples collected after rat slaughtering was determined using the protocol of Stefanov et al. [27]. In this context, 2-nitro-5-mercaptobenzoic acid, yellow colored, was formed by the reduction of GSH –SH from Ellman's reagent and sample centrifuged with 5% trichloroacetic acid to centrifuge proteins. Afterward, 0.1 mL of homogenate, 2 mL of phosphate buffer (pH 8.4), 0.5 mL of 5′5 dithiobis (2-nitrobenzoic acid), and 0.4 mL of double-distilled water were added. Afterward, the mixture was vortexed, and absorbance was measured using a spectrophotometer at 412 nm within 15 min, and the results were expressed as μmol/L.

The sodium oxide dismutase (SOD) activity was assessed following the protocol by [28]. For the analysis, the reaction mixture was composed of 1.2 mL of sodium pyrophosphate buffer (0.052 mM; pH 7.0) and 0.1 mL of phenazine methosulfate. Subsequently, 0.3 mL of supernatant obtained after centrifugation (1500 × g for 10 min followed by 10,000 g for 15 min) was introduced into reaction solution. The enzyme reaction was initiated by adding 0.2 mL of NADH (780 mM) and later halted by the addition of 1 mL of glacial acetic acid. The chromogen's amount was determined by observing the change in color intensity at 560 nm, and resulting values of SOD were expressed as units/g of tissue. Similarly, the catalase activity of the kidney samples was measured by following the protocol of [29], which is based on the dichromate reduction in acetic acid medium to chromic acetate, which is heated in the hydrogen peroxide presence to form (per-chromic acid) that is detected spectrophotometrically at an absorbance of 620 nm. Absorbance was taken at 620 nm after 1 min intervals against reagent blank.

### 2.12. Lipid Peroxidation in Kidney Tissues

The oxidation level of kidney tissue was determined by measuring malondialdehyde (MDA), a lipid peroxidation indicator, by following the protocol of [30]. Accordingly, 0.1 mL of processed tissue samples, acetic acid [1.5 mL (20%) pH 3.5], thiobarbituric acid [1.5 mL (0.8%)], and sodium dodecyl sulfate [0.2 mL (8.1%)] were added. Afterward, the mixture was heated for 60 min at 100°C, and after cooling with tap water, 5 mL of n-butanol: pyridine (15:1% v/v) and 1 mL of distilled water were added. Subsequently, the mixture was aggressively shaken followed by the removal of organic layer after 10 min of centrifugation at 4000 rpm, and then absorbance at 532 nm was measured with a spectrophotometer and the results were reported in nmol/g of tissue.

### 2.13. Histopathological Examination

The histopathological examination of rats' kidneys was done using a rotary micro-tome (MSLK233) following the procedure as explained by [31]. For the said parameter, kidney tissues were removed followed by placing them in formalin (10%) and then gradually dehydrated with ethanol (70%–100%) followed by washing in paraffin and xylene and embedding in paraffin wax. A rotary microtome was used to cut 5-mm thick sections, and then pieces were placed on cleaned slides. The paraffin wax-coated slides were put in slide holders and roasted at 40°C for 20 min to attach the tissue to them. After cooling for 30 min at room temperature, tissues were stained with hematoxylin for 6 min and eosin for 20 s. Afterward, tissue slices were dehydrated and arranged on slides with coverslips and DPX followed by placing slide specimens inspected under a light microscope for anomalies.

### 2.14. Statistical Analysis

Collected data were subjected to statistical analysis using the Statistical Package for the Social Sciences (SPSS) version 20 to assess the significance of differences among treatments by following the protocol of [32]. One-way analysis of variance (ANOVA) was used to investigate potential variations among study groups, and a post hoc analysis using Tuckey's test was performed for the comparison of means with significance level (*p* < 0.05), whereas the graph pad prism 8.1.4 was used for obtaining graphs of different study parameters.

## 3. Results

The study involves the measurement of the renal biomarkers, hematological examination, and IL-6 antioxidant enzymes of rats subjected to different levels of dietary lyophilized *Grewia asiatica* pulp powder. The details of the obtained results are discussed below.

### 3.1. Bioactive Profiling of *Grewia asiatica* Extracts Using Gas Chromatography–Mass Spectrometry

**Figure 1:**
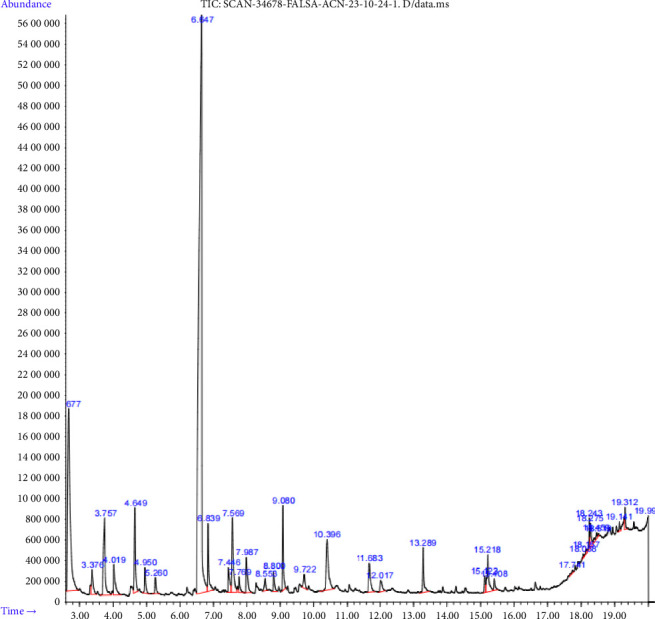
Bioactive compound chromatogram profile of the phalsa fruit pulp extract using GC–MS analysis.

Considering the results of the bioactive profiling of *Grewia asiatica* extracts using GC–MS, a total of 22 compounds were identified and a chromatograph of GC–MS is presented in (Figure 1), whereas the chemical compound, compound formula, retention time (min), molecular weight (g/mol), m/z ratio, and concentration in (%) are presented in (Table 2). The chemical compounds detected in phalsa fruit extract include 2,5-furandione 3-methyl, 2,5-furandione dihydro-3-methylene, thymine, 2-furancarboxylic acid, hydrazide, furyl hydroxymethyl ketone, 4H-pyran-4-one, 2,3-dihydro-3,5-dihydroxy-6-methyl, 2-propanamine, N-methyl-N-nitroso-,4-mercaptophenol, 1,3,5-benzenetriol, 2,4-difluoroanisole, 2-naphthalenol, phthalic anhydride, 3-butenoic acid, 3-methoxy-4-nitro, methyl ester, methyl 3-methoxydecanoate, 28-methyl-nonacosanoic acid, pyrrolidide, butanedioic acid, dipropyl ester, N-(2,2-dimethyl-propyl)-propionamide, D-allose, D-galactonic acid, gamma lactone, 3-deoxy-d-mannoic lactone, n-hexadecanoic acid, oleic acid, 9,12-octadecadienoic acid, and 9,12,15-octadecatrienoic acid.

### 3.2. Growth Parameters of Experimental Rats' Animal

The results for body and kidney weight of rats administered varying levels of lyophilized phalsa powder exhibited significant (*p* < 0.05) differences attributed to treatments and growth duration as depicted in Table 3. At the start of the experimental trial, rats' body weight ranged from 234.00 ± 7.79 g (*G*_1_) to 254.83 ± 14.97 g in *G*_4_ that were increased to 237.50 ± 17.75 g to 255.33 ± 15.83 g, respectively. At termination, the maximum body weight was recorded in G_3_ (262.83 ± 7.78 g) followed by G_4_ (257.50^b^ ± 11.11 g), while the minimum was observed in *G*_1_ (*rats given s*tandard diet along with casticin at a dose of 5 mg/kg body weight through oral gavage as well as CP at a dose of 5 mg/kg body weight). The *G*_2_ exhibited weight reduction at the termination compared to baseline, decreasing from 250.50 ± 10.58 g to 237.50 ± 33.80 g. Conversely, the body weight improved within *G*_3_, *G*_4_, *G*_5_, and *G*_6_, with maximum weight gain in *G*_3_, i.e., from 250.60 ± 10.58 g to 262.83 ± 7.78 g. The treatment group (*G*_4_) exhibited non-significant changes in body weight, transitioning from 254.00 ± 14.97 to 257.50 ± 11.11 g recorded on days 1^st^ and 33^rd^, respectively. The *G*_2_ (rats given diet along with CP at a dose of 5 mg/kg BW intraperitoneally) was the only experimental group that showed weight loss at the end of the experiment. In terms of kidney weight, rats belonging to group *G*_2_ exhibited the highest mean kidney weight compared to other experimental groups. The difference in kidney weight among other experimental groups was not statistically significant.

### 3.3. Serum Protein Levels as Biomarkers

**Figure 2:**
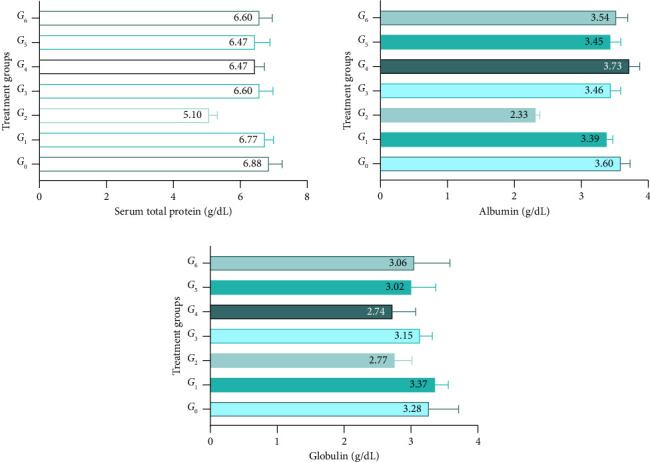
Serum total protein, albumin, and globulin levels of rats subjected to cisplatin injury and provided with different levels of lyophilized *Grewia asiatica* powder; *G*_0_: Rats given standard diet along intraperitoneal 0.9% saline solution; *G*_1_: Rats given standard diet along with casticin at a dose of 5 mg/kg body weight and cisplatin at a dose of 5 mg/kg body weight; *G*_2_: Rats given standard diet along with cisplatin (5 mg/kg BW) intraperitoneally; *G*_3_: Rats given standard diet along with injected intraperitoneal 0.9% saline solution and 400 mg/kg/day of lyophilized GA powder with distilled water; *G*_4_: Rats given standard diet along with 200 mg/kg/day of lyophilized GA powder and cisplatin (5 mg/kg. BW) intraperitoneally; *G*_5_: Rats given standard diet along with 300 mg/kg/day of lyophilized GA powder and cisplatin (5 mg/kg BW) intraperitoneally; *G*_6_: Rats given a standard diet along with 400 mg/kg/day of lyophilized GA powder and cisplatin (5 mg/kg BW) intraperitoneally.

The serum protein levels of rats subjected to CP nephropathy showed significant differences among treatments with different levels of lyophilized phalsa *powder* (*p* < 0.05). Means for total protein indicated a decline in *G*_2_ (positive control) as 5.10 ± 0.22 g/dL and an increase in *G*_5_ (rats given 300 mg *Grewia asiatica) as* 6.30 ± 0.24 g/dL. Furthermore, serum albumin and globulin levels reduced significantly in the nephrotoxic group of rats (*G*_2_) as 3.45 ± 0.34 and 3.02 ± 0.35 g/dL, respectively, while improvements were observed in treatment groups (*G*_5__&_*G*_6_) as 3.53 ± 0.38 and 3.06 ± 0.35 g/dL, respectively. The *G*_1_ (rats induced with CP and treated with casticin) showed non-significant changes in total protein, albumin, and globulin compared with the negative control as 6.77 ± 0.23, 3.69 ± 0.23, and 3.39 ± 0.18 g/dL (Figure 2).

### 3.4. Renal Function Test of Experimental Animal

Table 4 presents blood urea nitrogen (BUN) and creatinine level of rat serum provided with different levels of lyophilized *Grewia asiatica* fruit pulp powder as these parameters are critical in the assessment of renal impairment and gives valuable insight into the efficacy of therapeutic interventions. The BUN levels varied among groups, with the highest value recorded in *G*_2_ as 253.00 ± 0.37 mg/dL, indicating a severe increase in nitrogenous waste products in bloodstream. In contrast, *G*_0_ exhibited the lowest BUN at 45.00 ± 3.24 mg/dL, which is within the normal range. Notably, *G*_1_ and *G*_4_ showed elevated BUN levels of 76.00 ± 5.69 mg/dL and 64.00 ± 1.79 mg/dL, respectively, while *G*_5_ and *G*_6_ had lower values of 34.00 ± 0.87 mg/dL and 38.00 ± 4.38 mg/dL Statistical significance was observed, with *G*_2_ showing a significant elevation compared to all other groups (*p* < 0.01), whereas *G*_0_ and *G*_3_ presented the most comparable levels, indicating a stable renal function in those treatments. Similarly, creatinine levels also demonstrated significant variability, with *G*_2_ again reflecting the most critical condition at 4.90 mg/dL (±0.24). This value exceeds normal physiological limits and corroborates the elevated BUN levels, suggesting pronounced renal dysfunction. Conversely, *G*_0_ and *G*_3_ showed consistent creatinine levels at 0.50 ± 0.09 mg/dL, indicating normal renal clearance capabilities. *G*_1_ and *G*_4_ had elevated creatinine levels of 1.70 ± 0.14 mg/dL) and 1.00 ± 0.14 mg/dL, respectively, which may suggest mild to moderate renal impairment. In *G*_5_ and *G*_6_, creatinine levels of 0.60 ± 0.06 mg/dL and 0.70 ± 0.03 mg/dL indicate a relatively preserved renal function compared to *G*_2_, but still elevated compared to *G*_0_ and *G*_3_ as indicated in Table 4.

### 3.5. Hematological Profile Results

The hematological results (Table 5) comprising the RBC count, hemoglobin concentration, and HCT levels indicated a significant decline in values across treatment groups (*p* < 0.05). The control group (*G*_0_) shows a mean RBC count of 7.55 × 10^6^/μL, which is comparable to several other groups (*G*_1_, *G*_2_, and *G*_4_). *G*_3_ (8.67 × 10^6^/μL), *G*_5_ (9.34 × 10^6^/μL), and *G*_6_ (8.85 × 10^6^/μL) demonstrate significantly higher RBC counts compared to control (*G*_0_). This suggests that the treatments applied in *G*_3_, *G*_5_, and *G*_6_ may have stimulated erythropoiesis (the production of RBCs), improving the oxygen-carrying capacity of the blood. *G*_2_ (6.63 × 10^6^/μL) exhibited the lowest RBC count, which might indicate an impairment in RBC production or increased destruction (hemolysis) due to the use of CP. The control group (*G*_0_) shows a mean hemoglobin level of 13.00 g/dL, and groups *G*_1_, *G*_2_, and *G*_4_ display statistically similar values, ranging from 11.50 to 13.20 g/dL. The most notable increases were observed in *G*_3_ (15.90 g/dL), *G*_5_ (15.55 g/dL), and *G*_6_ (14.60 g/dL), showing significantly higher hemoglobin concentrations than the control. These results suggest enhanced oxygen transport efficiency in relevant groups, as hemoglobin binds and carries oxygen within the RBCs. In contrast, *G*_2_ (11.50 g/dL) demonstrates the lowest hemoglobin level, possibly reflecting a decline in the synthesis of hemoglobin or increased loss through bleeding or hemolysis. *G*_0_ exhibits a HCT level of 47.90%, which is statistically similar to the values found in *G*_1_ (43.30%), *G*_4_ (46.20%), and to some extent *G*_2_ (38.40%). The HCT is significantly higher in *G*_3_ (53.10%), *G*_5_ (56.00%), and *G*_6_ (54.00%), which correlates with the higher RBC counts and hemoglobin levels in these groups. A higher HCT suggests a greater proportion of RBCs in the blood volume. *G*_2_ (38.40%) shows the lowest HCT, which could indicate a state of anemia. The most substantial decrease was observed in *G*_2_ (nephrotoxic group) compared to control with RBC, hemoglobin, and HCT values. The treatments *G*_3_ and *G*_6_ reported improvements for RBCs, hemoglobin, and HCT levels to their maximum extent, underscoring the enhanced potential of phalsa dose at a dose of 400 mg/kg body weight in ameliorating serum profile compared to control displaying no apparent signs of toxicity. The hematological trends varied slightly in treatments *G*_4_ and *G*_5_, whereas significant improvements were observed in *G*_4_ followed by *G*_5_ compared to the control.

### 3.6. Antioxidant Enzyme Activity

Table 6 demonstrates the role of CP and subsequent treatment with *Grewia asiatica* lyophilized powder on the antioxidant defense system and lipid peroxidation in rats. The key parameters evaluated are MDA as a marker of lipid peroxidation, and the activity of key antioxidant enzymes: GSH, catalase, and superoxide dismutase (SOD). Antioxidant enzyme activities play a crucial role in counteracting oxidative stress and mitigating lipid peroxidation. MDA is a product of lipid peroxidation and serves as an indicator of oxidative damage to cell membranes. The variations were among MDA concentrations of different treatment groups, with the *G*_2_ showing the highest MDA level (47.78 nmol/g), indicating the most severe lipid peroxidation and oxidative stress in rats exposed to CP without any effective treatment. This suggests that CP has caused significant oxidative damage. Both *G*_0_ (control) and *G*_3_ show the lowest MDA levels (23.57 and 22.27 nmol/g, respectively), indicating less oxidative damage. *G*_3_, which received higher doses of Grewia asiatica and no CP, had levels statistically similar to the control. The *G*_1_, *G*_5_, and *G*_6_ exhibit moderate levels of MDA, ranging between 36.93 and 40.00 nmol/g, suggesting some level of oxidative protection by *Grewia asiatica* but not to the extent seen in *G*_3_. *G*_4_ (42.63 nmol/g) has relatively high MDA levels, indicating insufficient protection against lipid peroxidation compared to higher doses of *Grewia asiatica*. GSH is a vital antioxidant involved in neutralizing ROS and maintaining the redox balance within cells. *G*_2_ shows the lowest GSH levels (2.78 μmol/g), indicating a reduced antioxidant capacity, which correlates with the high oxidative damage observed through elevated MDA levels. Likewise, *G*_0_ (control) and *G*_3_ have significantly higher GSH levels (4.63 and 4.95 μmol/g, respectively), suggesting a well-maintained antioxidant defense. The high GSH levels in *G*_3_ suggest that *Grewia asiatica* dose at higher levels effectively enhances GSH production in normal rats, improving cellular protection. *G*_1_, *G*_5_, and *G*_6_ show moderate GSH levels ranging between 3.72 and 3.91 μmol/g, a partial improvement in antioxidant capacity. *G*_4_ has lower GSH levels (3.19 μmol/g) compared to the control and a higher *Grewia asiatica* dose, indicating that antioxidant protection is not as strong in this group. *G*_2_ and *G*_4_ have statistically higher (*p* < 0.05) GSH levels compared to *G*_1_. The enzyme SOD is critical in the detoxification of superoxide radicals, converting them into hydrogen peroxide, which is further neutralized by catalase. *G*_2_ exhibits the lowest SOD activity (5.68 U/g), reflecting severe oxidative stress and poor superoxide detoxification. *G*_0_ (control) and *G*_3_ have significantly higher SOD activity (11.66 and 12.2 U/g, respectively). The high SOD levels in *G*_3_ indicate enhanced superoxide neutralization, showing that *Grewia asiatica* increases the SOD activity. *G*_1_, *G*_5_, and *G*_6_ have moderate SOD levels, ranging between 8.07 and 8.80 U/g, suggesting the restoration of SOD activity in AKI-induced groups. *G*_4_ (7.59 U/g) shows a lower SOD activity compared to *G*_3_, further demonstrating that higher doses of Grewia asiatica are required for better oxidative protection. The SOD activity in *G*_2_ is significantly lower (*p* < 0.05) than in the control and *Grewia*-treated groups, especially *G*_3_, showing that oxidative damage in *G*_2_ is more pronounced due to insufficient superoxide neutralization. Catalase dissociates hydrogen peroxide (H_2_O_2_) into water and oxygen reducing oxidative stress. Also, the *G*_2_ shows the lowest catalase activity (23.87 U/g), corresponding with the highest oxidative stress and indicating severe impairment in antioxidant defenses. *G*_0_ (control) and *G*_3_ have significantly higher catalase activity, with *G*_3_ showing the highest activity (51.14 U/g). This indicates that Grewia asiatica enhances catalase activity, thereby improving the ability to neutralize hydrogen peroxide and reduce oxidative damage. *G*_1_, *G*_4_, *G*_5_, and *G*_6_ exhibit a moderate catalase activity, ranging from 30.62 to 44.17 U/g. There is a significant decrease in catalase activity in *G*_2_ compared to that of *G*_0_ and *G*_3_ (*p* < 0.05). *G*_3_ has the highest catalase activity, showing that *Grewia asiatica* powder can stimulate antioxidant enzymes in rats subjected to renal injury (Table 6).

### 3.7. Alanine Aminotransferase Level of Experimental Animal

**Figure 3:**
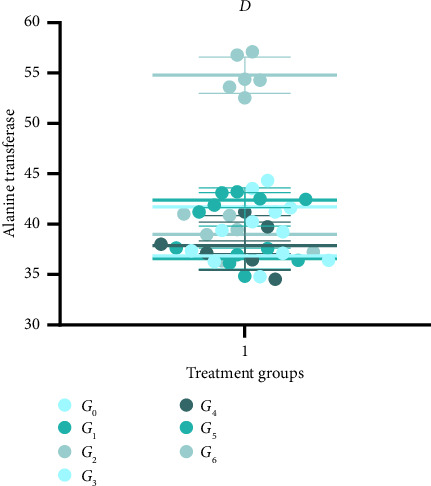
Alanine transferase level of rats subjected to cisplatin injury and provided with different levels of lyophilized *Grewia asiatica powder*.

The effect of dietary treatments on alanine aminotransferase (ALT) displayed statistically significant (*p* < 0.05) differences with highest value as 54.79 ± 1.80 IU/L observed in *G*_2_. However, the ALT values in treatment groups were 37.85 ± 2.37, 36.60 ± 1.05, and 38.98 ± 1.89 IU/L in *G*_4_, *G*_5_, and *G*_6_, respectively. There was a slight elevation for the said parameter in *G*_1_ and *G*_3_ than negative control with readings as 42.39 ± 0.75 and 41.72 ± 1.90 IU/L as reported in Figure 3.

The results regarding liver function test as indicated by alanine transferase (IU/L) level indicated significant (*p* < 0.05) results concerning treatments with a maximum peak observed in *G*_3_, which reduced in *G*_4_ and a minimum was observed in the *G*_6_ group as reported (Figure 3).

### 3.8. Interleukin-6 Level Estimations

**Figure 4:**
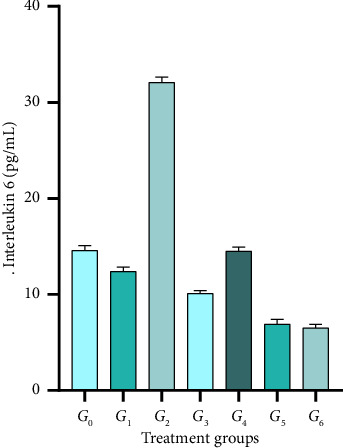
Interleukin-6 (pg/mL) of rats subjected to cisplatin injury and provided with different levels of lyophilized *Grewia asiatica* powder; *G*_0_: Rats given a standard diet along intraperitoneal 0.9% saline solution; *G*_1_: Rats given a standard diet along with casticin at a dose of 5 mg/kg body weight and cisplatin at a dose of 5 mg/kg body weight; *G*_2_: Rats given a standard diet along with cisplatin (5 mg/kg BW) intraperitoneally; *G*_3_: Rats given a standard diet along with injected intraperitoneal 0.9% saline solution and 400 mg/kg/day of lyophilized GA powder with distilled water; *G*_4_: Rats given a standard diet along with 200 mg/kg/day of lyophilized GA powder and cisplatin (5 mg/kg BW) intraperitoneally; *G*_5_: Rats given a standard diet along with 300 mg/kg/day of lyophilized GA powder and cisplatin (5 mg/kg BW) intraperitoneally; *G*_6_: Rats given a standard diet along with 400 mg/kg/day of lyophilized GA powder and cisplatin (5 mg/kg BW) intraperitoneally.

IL-6 is a cytokine with a small protein that plays a pivotal role in cell signaling and immune responses. It is produced by different body cells, especially immune cells, and helps regulate inflammation, immune responses, and hematopoiesis. The elevated levels of IL-6 are often associated with inflammation and diseases, including autoimmune disorders, certain cancers, and infectious diseases. The results regarding the IL-6 level of rats' blood serum subjected to CP-induced AKI along with dietary lyophilized Grewia asiatica powder showed statistically significant (*p* < 0.05) differences (Figure 4). Means reported the highest value of 32.1 ± 0.56 pg/mL in *G*_2_ (rats given standard diet along with CP (5 mg/kg body weight) intraperitoneally) and lowest as 6.5 ± 0.38 pg/mL in *G*_6_ Rats given standard diet along with 400 mg/kg/day of lyophilized GA powder and CP (5 mg/kg body weight). Results elaborated non-significant differences between *G*_1_ and *G*_3_ compared to negative control with values as 12.4 ± 0.45 and 10.1 ± 0.43 pg/mL, respectively. Contrary to this, IL-6 values decreased substantially in *G*_5_ and *G*_6_ as 6.9 ± 0.38 and 6.5 ± 0.49 pg./mL, respectively. The results indicated that dietary intervention comprising 300 and 400 mg/kg of body weight significantly improved inflammation status in nephropathic rats.

The results regarding pro-inflammatory IL-6 indicated significant (*p* < 0.05) variation results (Figure 4), showing that the maximum peak was observed in *G*_2_, which was reduced in *G*_3_. However, it slightly increased in *G*_4_ but dropped significantly in *G*_5_ and *G*_6_ as indicated.

### 3.9. Histopathological Changes in Renal Tissues of Rats

**Figure 5:**
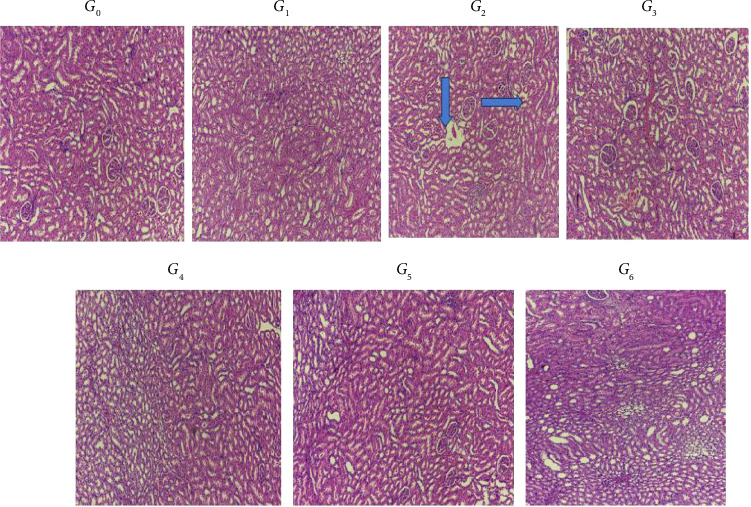
Histopathological changes in renal tissues of rats subjected to cisplatin injury and provided with different levels of lyophilized *Grewia asiatica* powder. *G*_0_: Rats given a standard diet along intraperitoneal 0.9% saline solution; *G*_1_: Rats given a standard diet along with casticin at a dose of 5 mg/kg body weight and cisplatin at a dose of 5 mg/kg body weight; *G*_2_: Rats given a standard diet along with cisplatin (5 mg/kg body weight) intraperitoneally; *G*_3_: Rats given a standard diet along with injected intraperitoneal 0.9% saline solution and 400 mg/kg/day of lyophilized GA powder with distilled water; *G*_4_: Rats given a standard diet along with 200 mg/kg/day of lyophilized GA powder and cisplatin (5 mg/kg BW) intraperitoneally; *G*_5_: Rats given a standard diet along with 300 mg/kg/day of lyophilized GA powder and cisplatin (5 mg/kg BW) intraperitoneally; *G*_6_: Rats given a standard diet along with 400 mg/kg/day of lyophilized GA powder and cisplatin (5 mg/kg BW) intraperitoneally.

The results (Figure 5) showed H&E-stained sections of the paraffin-embedded kidney tissues of rats subjected to CP-induced AKI. The histopathology examination of the rat kidney showed kidney tissues of rats from group *G*_0_ normal and organized histoarchitecture with intact glomerulus and renal tubules. Also, rats were treated with a single intraperitoneal injection of CP at a dose 5 mg/kg body weight on the 28th day of study and sacrificed at the termination of the study point. Hematoxylin and eosin staining of *G*_2_ revealed that normal glomeruli but slightly widened tubular parts, as well as the dilation of renal tubules, was observed, indicating tubular injury. The kidney tissue section of rats of all other groups, *G*_3_, *G*_4_, *G*_5_, and *G*_6_, does not show evident tubular injury.

## 4. Discussion

Phalsa fruit, renowned for its myriad health benefits, is widely embraced as a therapeutic intervention for promoting human health. The phytochemical screening of acetonitrile fraction by GCMS revealed the presence of several bioactive components like 2,5-furandione, 3-methyl, 2,5-furandione, and dihydro-3-methylene that are citric acid derivates and are also present in lime juice and responsible for anti-bacterial activity [33]. Similarly, another study reported that 4-mercaptophenol was also identified in grapes and has many health benefits [34]. Also, another study stated that furyl hydroxymethyl ketone and 4H-pyran-4-one, 2,3-dihydro-3,5-dihydroxy-6-methyl possess anti-bacterial anti-inflammatory and anti-cancer activities [35, 36]. In this research study, nephroprotective effect and safety profile of *Grewia asiatica* were assessed in Wistar albino rats over a period of 33 days. The rats were administered CP at a dose of 5 mg/kg, which is primarily utilized in neoplasm treatment but known to induce nephrotoxicity. The study encompassed an array of parameters including body weight, renal function, liver function tests (LFTs), IL-6 levels, and antioxidant enzymes along with hematological parameters during the study period.

Throughout the study duration, no signs of toxicity were observed in the rats, and they remained healthy and survived within the stipulated safety dose. Body weight and relative kidney weight are some of the indicators of the renal damage [37]. Correspondingly, nephropathic rats exhibited decreased body weight. These findings are consistent with results reported by [38], indicating weight reduction in response to renal damage-induced stress and increased kidney weight. According to [39], there is a decreased gut motility, thus reducing food intake resulting in weight loss. Additionally, CP administration was associated with muscle wasting and weight loss as reported in previous studies. Previous literature on CP-induced complications has elucidated mechanisms involving metabolism alteration, fat catabolism, and cachexia, alongside factors like oxidative stress, cytokine excess, up-regulation, and modification of ubiquitin–proteasome system [40, 41]. However [35], in the present study, treatments provided with lyophilized phalsa powder exhibited improved body weight, underscoring potential of phalsa due to the presence of reno-protective bioactive components like 9,12,15-octadecatrienoic acid, (Z,Z,Z) as indicated by El Sawi et al. [42] in alleviating CP-induced side effects. Moreover, no significant variation in body weight was noted in the safety group, indicating the absence of adverse effects on rats. These findings highlight the promising therapeutic properties of lyophilized phalsa fruit and its potential as intervention for mitigating adverse effects of CP therapy. Reference [43] mentioned that the weight of rats was stably treated with phytochemicals; the same results were indicated by [44] with the use of fruit rich in phytochemicals as a therapeutic agent against nephropathy.

The results of this study revealed an augmentation in lipid peroxidation and reduction of antioxidant enzymes. Several theories have emerged about the role of structural and functional anomalies in the development of inflammatory and oxidative stress in renal tissues [45]. CP is responsible for oxidative stress and lipid peroxidation through multiple mechanisms as the accumulation of CP in the kidney results in a reduction of tubular, vascular, and glomerular functions [46]. CP triggers numerous cytotoxic mediators that enhance renal tubular cell death and activates the FAS ligand system, which is one of the key regulators of renal tubular apoptosis and oxidative stress. Thus, renal mFasL and mFas expressions cause multiplicative oxidative stress-induced renal damage [47]. This has been proved that lipid peroxidation and oxidative stress are associated with 8-OhdG and MDA; in addition to these, lipid peroxidation also generates prostaglandin F2*α* isomer F_2_-isoprostanes (F_2_-IsoPs). The production of these oxidative mediators acts in the modification of protein and nucleic acid, resulting in the synthesis of aldehyde and aldehyde-proteins, which ultimately disturb the normal physiological functions and promote multiple metabolic disorders [48]. The oxidative stress and lipid peroxidation were reduced in treatment groups, proving antioxidant potential of *Grewia asiatica* as reported by [49], who stated the in vitro antioxidant potential of phalsa bark as indicated by the DPPH assay [50]. Curcumin and flax seed when given to rats with CP induced AKI; both improved the ROS status compared to CP only group [51].

The oxidative stress triggers inflammatory biomarkers; therefore, IL-6 is augmented in nephrotoxic rats as IL-6 is a pro-inflammatory cytokine, involved in the progression of inflammation. IL-6 is a master cytokine that promotes inflammation and activates acute phase protein. The inflammation triggers other inflammatory factors like TNF-α and advances via NF-κB and MAPK pathways. The NF-κB family contains five transcription factors, i.e., RelA (p65), P50, c-Rel, p52, and RelB, and these transcription factors are regulated by IκB proteins. However, free radicals and oxidative stress alter these factors as well as promote pro-inflammatory expression [52]. Our study reported dropped levels of IL-6 in the treatment group; this might be due to the presence of 9-octadecenoic acid in phalsa, which modifies the MAPKs and NF-κB responses to control inflammation [53] and the results are supported by the study of [54], who worked on fruit-derived bioactive compounds and their anti-inflammatory potential. They reported that serum albumin and globulin levels dropped as inflammation results in the form of vasodilation, allowing serum proteins to escape from blood vessels [55]. However, inflammation and vasodilation reduced while serum protein improved in treatment groups supplemented with lyophilized phalsa. These findings correlate with results of [56] where the hepatoprotective activity of berberine in rats reduced inflammation while improving protein profile. Similarly, [57] worked on casticin to evaluate its role in the amelioration of inflammation via NF-ĸB in pulmonary disease and reported similar findings.

The oxidative stress and lipid peroxidation lead to other complications; thus, results of RFT (BUN and serum creatinine) significantly increased in rats injected with CP proving its nephrotoxic effects [58]. A wide array of mechanisms and factors responsible for CP-induced nephropathy increased MDA levels and decreased cellular GSH levels, leading to lipid peroxidation and DNA damage. Besides this, studies proved that CP also reduced cellular catalase and GSH peroxidase; a reduction of these enzymes promotes the production of free radical species in renal cells, which assist in disease progression [59, 60]. The other mechanism in pathological manifestation is the necrosis of proximal renal tubular epithelial cells and dysfunction of cytoplasmic organelle, specifically in the mitochondria and endoplasmic reticulum [61]. The reported alterations in organelles are an expansion of endoplasmic reticulum, proximal tubule dilation, amplified numbers of lysosomes, and swollen mitochondria [62]. Similarly, [63] reported CP hydrate, a process carried out through the accumulation of CP in renal tubular cells by cation transporter 2 (OCT2); thus, this mechanism leads to oxidative stress and nephron damage. However, the supplementation of *Grewia asiatica* attenuates nephropathy in treatment groups. Studies have proved that *Grewia asiatica* contains a broad variety of bioactive compounds acting as antioxidants, involved in reducing oxidative damage and providing aid to recover renal function. The results of improved renal function were supported by the reports of [13]; and [64], which stated the antioxidant, anti-inflammatory, and nephroprotective effects of *Grewia asiatica*.

The elevated ALT levels of rats' blood indicated hepatic inflammation because liver is the chief organ involved in the metabolism of xenobiotics. Chemotherapy with CP induce hepatotoxicity and nephrotoxicity [65]; in addition to this, oxidative stress, inflammation, and nephropathy are also responsible for hepatotoxicity and augmented liver enzymes. The accumulation of CP in the liver is accompanied by the induction of apoptosis via intrinsic caspases, activation of tumor-suppressor protein p53, increased MDA, positive caspase-3 reactions, and loss of liver tissue structure [66, 67]. The supplementation of *Grewia asiatica* reduced ALT levels and improved hepatic function; the results are in harmony with the findings of [68] research on oxidative stress mechanisms and gene expressions in diabetic neuropathy and reported that the reduction of these genes alleviates oxidative stress and organ damage. Reference [43] observed the *Ziziphus spina-christi* when administered to rats with CP induced hepato-renal damage, significantly improving both hepatic and renal functions.

The reduced RBC, hemoglobin, and HCT are the result of nephropathy and reduced erythropoietin (EPO), as kidneys are responsible for the synthesis of EPO, a hormone involved in hematopoiesis [69]. The ROS species produced due to CP toxicity reduce the RBC count [70]. The supplementation of *Grewia asiatica* improved RBC and hemoglobin in treatment groups, as the phytochemicals improved renal function and EPO. In addition, vitamin C is a major component of *Grewia asiatica* involved in the synthesis of hemoglobin. The outcomes of improved hematological profile were advocated by the results of [71] hemoglobin and ferritin in pregnant females.

## 5. Conclusion

Our study results indicated that lyophilized *Grewia asiatica* pulp powder has nephroprotective properties as indicated by the rat model of CP-induced AKI. The observed improvements in body and kidney weight, serum protein, renal function and hematological parameters, antioxidant activity, liver function, and inflammatory indicators demonstrate that *Grewia asiatica* has multifaceted protective properties. Overall, 300 mg/kg of lyophilized phalsa powder exhibited a noteworthy protective effect against CP-induced nephropathy and finding suggests the use of GA powder as a promising management strategy for mitigating nephrotoxicity in the clinical setup. These findings call for further investigation of the underlying processes and their translation into therapeutic applications for the management of AKI leading toward kidney health protection. Additionally, comparative analysis with casticin provides useful information on the relative efficacy of natural remedies, opening door for future therapeutic approaches in the management of renal disease.

## Figures and Tables

**Table 1:** Study plan for the experiment trial.

Treatments	Description
G_0_	Rats given standard diet along with intraperitoneal 0.9% saline solution
G_1_	Rats given *s*tandard diet along with casticin at a dose of 5 mg/kg body weight through oral gavage as well as cisplatin at a dose of 5 mg/kg body weight
G_2_	Rats given standard diet along with cisplatin (5 mg/kg BW) intraperitoneally
G_3_	Rats given standard diet along with injected intraperitoneal 0.9% saline solution and 400 mg/kg/day lyophilized GA powder orally with distilled water
G_4_	Rats given standard diet along with 200 mg/kg/day of lyophilized GA powder orally and cisplatin (5 mg/kg. BW) intraperitoneally
G_5_	Rats given standard diet along with 300 mg/kg/day of lyophilized GA powder orally and cisplatin (5 mg/kg BW) intraperitoneally
G_6_	Rats given a standard diet along with 400 mg/kg/day of lyophilized GA powder orally and cisplatin (5 mg/kg BW) intraperitoneally

**Table 2:** Bioactive compound profile of the phalsa fruit extract using gas chromatography–mass spectrometry.

No	Compound	Formula	Retention time (min)	Molecular weight (g/mol)	m/z ratio	%
1	2,5-furandione, 3-methyl	C_5_H_4_O_3_	2.67	112.08	68.9	0.77
2	2,5-furandione, dihydro-3-methylene	C_5_H_6_O	2.68	114.09	53	0.39
3	Thymine	C_9_H_10_N_2_O_3_	3.757	194.18	55.10	3.80
4	2-furancarboxylic acid, hydrazide	C_5_H_6_N_2_O_2_	4.019	126.11	126.10	1.51
5	Furyl hydroxymethyl ketone	C_6_H_6_O_3_	4.019	126.11	96.00	1.51
6	4H-pyran-4-one, 2,3-dihydro-3,5-dihydroxy-6-methyl	C_6_H_8_O	4.649	144.12	101.10	2.81
7	2-propanamine, N-methyl-N-nitroso-	C_4_H_10_N_2_O	5.260	102.13	61.10	0.59
8	4-mercaptophenol	C_6_H_6_OS	6.839	126.17	73.10	1.94
9	1,3,5-benzenetriol	C_6_H_6_O_3_	7.446	126.11	97.10	1.04
10	2-naphthalenol	C_10_H_8_O	7.769	144.16	126.10	0.56
11	Phthalic anhydride	C_8_H_4_O_3_	7.987	148.10	76.10	1.55
12	Methyl 3-methoxydecanoate	CH_3_(CH2)_8_COOCH_3_	8.553	186.29	101.00	0.50
13	28-methyl-nonacosanoic acid, pyrrolidide	C_16_H_32_O_2_	8.80	256.42	57.10	0.66
14	Butanedioic acid, dipropyl ester	C_10_H_18_O_4_	9.08	202.24	55.10	2.27
15	N-(2,2-dimethyl-propyl)-propionamide	C_8_H_17_NO	9.722	143.23	58.10	0.64
16	D-allose	C_6_H_12_O_6_	10.396	180.16	57.10	3.33
17	D-galactonic acid, Gamma-lactone	C_6_H_10_O_6_	11.683	178.14	61.00	1.66
18	3-deoxy-d-mannoic lactone	C_6_H_10_O	12.017	162.14	102.10	0.69
19	n-hexadecanoic acid	C_16_H_32_O	13.289	256.42	60.00	1.54
20	Oleic acid	C_18_H_34_O_2_	15.123	282.46	69.10	0.47
21	9,12-octadecadienoic acid	C_18_H_32_O	15.321	280.44	81.10	0.43
22	9,12,15-octadecatrienoic acid	C_18_H_30_O	15.408	278.42	82.10	0.44

**Table 3:** Effect of different treatments on body and kidney weight of rats subjected to dietary intervention of phalsa lyophilized powder.

Treatments	Rats weight (baseline vs. termination)		Kidney weight at trial termination
	Day 1	Day 33	
*G*_0_	240.67 ± 4.08	251.67 ± 7.42	0.71^b^ ± 0.02
*G*_1_	234.02 ± 7.79	237.50 ± 17.75	0.74^b^ ± 0.14
*G*_2_	250.50 ± 10.58	237.50 ± 33.80	1.04^a^ ± 0.06
*G*_3_	250.60 ± 10.58	262.83 ± 7.78	0.70^b^ ± 0.02
*G*_4_	254.83^a^ ± 14.97	257.50 ± 11.11	0.78^b^ ± 0.05
*G*_5_	248.50 ± 18.78	255.50 ± 23.78	0.71^b^ ± 0.05
*G*_6_	249.83 ± 22.26	255.33 ± 15.83	0.72^b^ ± 0.03

*Note:* Data are means ± SD. All values within the same column that have different letters in superscript are considered significantly different (*p* < 0.05). *G*_0_ = Negative control in which rats given standard diet along with intraperitoneal 0.9% saline solution, *G*_1_ = Positive control in which rats given standard diet along with casticin at a dose of 5 mg/kg body weight + CP at a dose of 5 mg/kg body weight, *G*_2_ = Rats given diet along with CP (5 mg/kg BW), *G*_3_ = Rats given standard diet + intraperitoneal 0.9% saline solution + 400 mg/kg/day lyophilized GA powder, *G*_4_ = Rats given diet + 200 mg/kg/day lyophilized GA powder + CP (5 mg/kg BW), *G*_5_ = Rats given diet + 300 mg/kg/day lyophilized GA powder + CP (5 mg/kg BW, *G*_6_ = Rats given diet + 400 mg/kg/day lyophilized GA powder + CP (5 mg/kg BW).

**Table 4:** Renal function tests of experimental rats subjected to cisplatin-based renal injury and provided with different levels of *Grewia asiatica* lyophilized powder.

Treatments	Blood urea nitrogen (mg/dL)	Creatinine (mg/dL)
*G*_0_	45.00^d^ ± 3.24	0.50^e^ ± 0.09
*G*_1_	76.00^b^ ± 5.69	1.70^b^ ± 0.14
*G*_2_	253.00^a^ ± 0.37	4.90^a^ ± 0.24
*G*_3_	28.00^f^ ± 1.67	0.50^e^ ± 0.09
*G*_4_	64.00^c^ ± 1.79	1.00^c^ ± 0.14
*G*_5_	34.00^e^ ± 0.87	0.60^d^ ± 0.06
*G*_6_	38.00^e^ ± 4.38	0.70^d^ ± 0.03

*Note:* Data are means ± SD. All values within the same column that have different letters in superscript are considered significantly different (*p* < 0.05). *G*_0_ = Negative control in which rats given standard diet along with intraperitoneal 0.9% saline solution, *G*_1_ = Positive control in which rats given standard diet along with casticin at a dose of 5 mg/kg body weight + CP at a dose of 5 mg/kg body weight, *G*_2_ = Rats given diet along with CP (5 mg/kg BW), *G*_3_ = Rats given standard diet + intraperitoneal 0.9% saline solution + 400 mg/kg/day of lyophilized GA powder, *G*_4_ = Rats given diet + 200 mg/kg/day of lyophilized GA powder + CP (5 mg/kg BW), *G*_5_ = Rats given diet + 300 mg/kg/day of lyophilized GA powder + CP (5 mg/kg BW, *G*_6_ = Rats given diet + 400 mg/kg/day of lyophilized GA powder + CP (5 mg/kg BW).

**Table 5:** Hematological profile including red blood cells, hemoglobin, and hematocrit of experimental rats subjected to cisplatin-based renal injury and provided with different levels of *Grewia asiatica* lyophilized powder.

Treatments	Red blood cell count (×10^6^/μL)	Hemoglobin (g/dL)	Hematocrit (%)
*G*_0_	7.55^b^ ± 0.25	13.00^b^ ± 0.83	47.90^c^ ± 0.91
*G*_1_	6.97^b^ ± 0.21	12.60^b^ ± 0.43	43.30^c^ ± 0.81
*G*_2_	6.63^b^ ± 0.31	11.50^c^ ± 0.33	38.40^d^ ± 0.46
*G*_3_	8.67^a^ ± 0.14	15.9.0^a^ ± 0.93	53.10^a^ ± 0.63
*G*_4_	7.88^b^ ± 0.56	13.20^b^ ± 0.94	46.20^c^ ± 1.88
*G*_5_	9.34^a^ ± 0.27	15.55^a^ ± 0.27	56.00^a^ ± 3.16
*G*_6_	8.85^a^ ± 0.74	14.60^a^ ± 0.55	54.00^ab^ ± 3.58

*Note:* Data are means ± SD. All values within the same column that have different letters in superscript are considered significantly different (*p* < 0.05). *G*_0_ = Negative control in which rats given a standard diet along with intraperitoneal 0.9% saline solution, *G*_1_ = Positive control in which rats given a standard diet along with casticin at a dose of 5 mg/kg body weight + CP at a dose of 5 mg/kg body weight, *G*_2_ = Rats given diet along with CP (5 mg/kg BW), *G*_3_ = Rats given standard diet + intraperitoneal 0.9% saline solution + 400 mg/kg/day of lyophilized GA powder, *G*_4_ = Rats given diet + 200 mg/kg/day of lyophilized GA powder + CP (5 mg/kg BW), *G*_5_ = Rats given diet + 300 mg/kg/day of lyophilized GA powder + CP (5 mg/kg BW, *G*_6_ = Rats given diet + 400 mg/kg/day of lyophilized GA powder + CP (5 mg/kg BW).

**Table 6:** Antioxidant enzymes including catalase, sodium oxide dismutase, and glutathione (GSH) levels along with lipid peroxidation of experimental rats subjected to cisplatin-based renal injury and provided with different levels of *Grewia asiatica* lyophilized powder.

Treatments	Malonaldehyde (nmol/g)	GSH (μmol/g)	Catalase (U/g)	Sodium oxide dismutase (U/g)
*G*_0_	23.57^d^ ± 1.47	4.63^c^ ± 0.50	48.56^d^ ± 1.30	11.66^d^ ± 0.25
*G*_1_	37.40^c^ ± 0.80	3.72^b^ ± 0.15	30.62^b^ ± 1.59	8.40^c^ ± 0.83
*G*_2_	47.78^a^ ± 2.50	2.78^a^ ± 0.19	23.87^a^ ± 1.46	5.68^a^ ± 0.54
*G*_3_	22.27^d^ ± 2.00	4.95^c^ ± 0.47	51.14^e^ ± 1.82	12.22^d^ ± 0.81
*G*_4_	42.63^b^ ± 2.17	3.19^a^ ± 0.16	34.46^c^ ± 1.63	7.59^bc^ ± 0.94
*G*_5_	36.93^c^ ± 1.76	3.91^b^ ± 0.25	41.38^d^ ± 1.25	8.07^b^ ± 0.84
*G*_6_	40.00^b^ ± 1.01	3.86^b^ ± 0.57	44.17^d^ ± 0.92	8.80^bc^ ± 1.27

*Note:* Data are means ± SD. All values within the same column that have different letters in superscript are considered significantly different (*p* < 0.05). *G*_0_ = Negative control in which rats given a standard diet along with intraperitoneal 0.9% saline solution, *G*_1_ = Positive control in which rats given a standard diet along with casticin at a dose of 5 mg/kg body weight + CP at a dose of 5 mg/kg body weight, *G*_2_ = Rats given diet along with CP (5 mg/kg BW), *G*_3_ = Rats given standard diet + intraperitoneal 0.9% saline solution + 400 mg/kg/day of lyophilized GA powder, *G*_4_ = Rats given diet + 200 mg/kg/day of lyophilized GA powder + CP (5 mg/kg BW), *G*_5_ = Rats given diet + 300 mg/kg/day of lyophilized GA powder + CP (5 mg/kg BW, *G*_6_ = Rats given diet + 400 mg/kg/day of lyophilized GA powder + CP (5 mg/kg BW).

## Data Availability

The datasets used and/or analyzed in the present study are available from the corresponding author upon reasonable request.
